# The German hearing in noise test with a female talker: development and comparison with German male speech test

**DOI:** 10.1007/s00405-023-07820-5

**Published:** 2023-01-12

**Authors:** Anna-Lena Mönnich, Sebastian Strieth, Andrea Bohnert, Benjamin Philipp Ernst, Tobias Rader

**Affiliations:** 1 Department of Otorhinolaryngology, University Medical Center Mainz, Mainz, Germany; 2 Department of Otorhinolaryngology, University Medical Center Bonn (UKB), Bonn, Germany; 3 Division of Audiology, Department of Otorhinolaryngology, University Hospital LMU Munich, Munich, Germany; 4 Abteilung Audiologie, LMU Klinikum, Klinik für Hals-Nasen-Ohrenheilkunde, Marchioninistr. 15, 81377 Munich, Germany

**Keywords:** Speech test, HINT, Hearing in noise test, Speech perception, Normal hearing, Speech reception threshold

## Abstract

**Purpose:**

The aim of the study was to develop the German Hearing in Noise Test (HINT) with female speaker by fulfilling the recommendations by International Collegium of Rehabilitative Audiology (ICRA) for using a female speaker to create new multilingual speech tests and to determine norms and to compare these norms with German male speech tests—the male speakers HINT and the Oldenburg Sentence Test (OLSA).

**Methods:**

The HINT with a female speaker consists of the same speech material as the male speaking HINT. After recording the speech material, 10 normal hearing subjects were included to determine the performance–intensity function (PI function). 24 subjects were part of the measurements to determine the norms and compare them with the norms of male HINT and OLSA. Comparably, adaptive, open-set methods under headphones (HINT) and sound field (OLSA) were used.

**Results:**

Acoustic phonetic analysis demonstrated significant difference in mean fundamental frequency, its range and mean speaking rate between both HINT speakers. The calculated norms by three of the tested four conditions of the HINT with a female speaker are not significantly different from the norms with a male speaker. No significant effect of the speaker’s gender of the first HINT measurement and no significant correlation between the threshold results of the HINT and the OLSA were determined.

**Conclusions:**

The Norms for German HINT with a female speaker are comparable to the norms of the HINT with a male speaker. The speech intelligibility score of the HINT does not depend on the speakers’ gender despite significant difference of acoustic–phonetic parameters between the female and male HINT speaker’s voice. Instead, the speech intelligibility rating must be seen as a function of the used speech material.

## Introduction

Speech audiometric tests are part of audiological diagnostic to assess speech intelligibility, to diagnose hearing ability and to evaluate communication handicaps under realistic conditions. The German speech audiometric tests Freiburger Speech Test (Hahlbrock 1953) [12], Göttinger Sentence Test (Kollmeier and Wesselkamp 1997) [21], and OLSA (Wagener, Brand and Kollmeier 1999) [33–35] are important parts in audiological rehabilitation and fitting process of various hearing devices.

In addition to the word recognition score (WRS), the target value of the speech test is the language reception threshold (SRT). The SRT can be measured via fixed or adaptive protocol, via headphones or sound field and in quiet or noisy conditions. Due to the spatial separation of speech and noise source, it is possible to evaluate the Intelligibility Level Difference (ILD) and Binaural Intelligibility Level Difference (BILD) and to assess benefits by bilateral hearing aid fitting. Binaural test conditions are preferred, because most people hear binaurally and speech and noise signals are often spatially separated in daily acoustic environment. The resulting HINT thresholds are influenced by interaural time and level differences in conditions with spatial separation. Nevertheless, the objective of this study was about the female HINT, it’s evaluation and comparison with the male HINT and not to assess the effects of binaural hearing [7, 26].

The American English HINT was developed in 1994 by Nilsson, Soli and Sullivan [26]. The HINT is used to evaluate the impact of hearing impairment on communication and to review speech in noise performance in the context of hearing aid users [14, 26]. The speech material consists of 12 lists, each containing 20 short, everyday, natural sentences, each spoken by a male speaker. The German HINT with a male speaker was developed by Joiko et al. in 2020 [17]. The HINT can be used from 6 years and older because of its simple grammatical structure and vocabulary. Like OLSA, two lists for training procedure should be included in the test protocol (Hällgren et al.). The test should provide reliable assessment of speech intelligibility in noise by standardized conditions. Standardized norms of the HINT are evaluated for 24 languages [17, 26, 30].

The noises by OLSA and HINT were both determined by multiple overlapping with used speech material resulting in noise that was fully spectrally matched to the sentence (Nilsson and Wagener). The equality of the long-term average speech spectrum (LTASS) for 12 languages, including English and German was shown by Byrne et al. in 1994. Furthermore, the authors shown that the LTASS of female and male voices agree within 2 dB over the frequency range from 250 to 5000 Hz, which is assumed to be the main level of everyday speech (Byrne et al. 1994).

The Performance intensity (PI) function is used to describe the correlation of the intelligibility of the test items depending on different sound pressure levels or SNRs in percent per dB. The steeper the slope, the more accurate the SRT results and the more sensitive the test procedure. The slope of the PI function by OLSA is about 17.1% per dB [33].

Soli and Wong (2008) compared the HINT norms of 13 languages. The slopes of the PI function were in the same range across all languages, despite speaker specific masking noise. The mean slope was about 10.3% per dB and the SD about 1.5 dB across all languages [30].

In 2015 Akeroyd et al. created on behalf of ICRA recommendations for development of new multilingual speech tests. The selected speaker should have normal articulating, acceptable and neutral dialect, does not need to be formally trained, should be able to control the vocalization effort during the recording session and should be female. The female voice seems to be an “acoustic compromise” between male and children’s speech [2]. The speaking fundamental frequency (F0) depends on factors, such as average vocal tract size, membranous length of vocal fold and amplitude of vibration. The mean F0 is around 100 till 120 Hz for male talkers and around 200 and 220 Hz for female talkers [31].

Few studies have developed and evaluated speech audiometric tests with female speakers (e.g., German bisyllable rime test in noise by Kliem and Kollmeier 1994 [19], sentence test by Bradlow et al. 1996 [5], Swedish HINT by Larsby et al. 2015 [23] and bilingual OLSA by Hochmuth et al. 2015 [16]. The Authors analyzed and discussed the influence of the speaker’s gender on acoustic–phonetic parameters, e.g., speaking rate, mean F0 or vowel space. Due to different test variables, such as speech material, interfering noise, test protocol and individual acoustic–phonetic parameters, standardized norms and general statements about the clear link between speakers’ gender, acoustic–phonetic parameters and the SRT are not available. The HINT within its realistic representation of daily communications environment, is favorable to the sometimes meaningless and limited sentence and vocabulary of matrix tests because of its simple, natural structure and up-to-date vocabulary [25, 34–36]. The consequence was the development of German HINT with female speaker. The evaluated SRT norms are generally the first norms using a female speaker in any HINT language. Finally, the norms of all HINT languages are easy comparable using calculated percentiles and the H-Score [30].

## Materials and methods

### Subjects

A group of 34 native German-speaking adults between 23 and 41 years (mean ± SD 26.8 ± 3.4 years) were recruited. Ten of them were participants to evaluate the PI function, 24 were participants to determine the norms. Subjects were screened for normal hearing using pure-tone hearing threshold levels ≤ 20 dB hearing level for frequencies 250–8000 Hz (defined by current ISO standard for normal hearing thresholds, ISO DIN 7029:2017-06) [10]. Participants with any audiological diseases or hearing disorders were excluded. All subjects were informed about the study and needed to sign informed consent before participating.

### Experimental setup

The recording and testing took place in IAC acoustics soundproof audiology test room at the University Medical Center Mainz. The recording was done using ½-inch free field ACO Pacific microphone, NTI Audio MA220 preamplifier and RME Fireface UC 24-bit 44 kHz sound card for transferring the audio to the computer.

The speech intelligibility tests were conducted using the OLSA Study Software 1.3 Oldenburg Mess program (HörTech gGmbH, Oldenburg, Germany) and the HNIT Software Hearing Test Device (Version 1.2, House Ear Institute, Los Angeles, California and Bio-logic System Corp, Mundelein, Illinois, US). The norms were determined using a computer equipped with a high-quality audio 24-bit, 18-channel digital-to-analog converter (RME Fireface UC).

### OLSA

The OLSA matrix sentence test was used to compare the results of the HINT with the clinical established and standardized male speech-in-noise-test. An “open-set” procedure in the sound field with noise and speech from the same loudspeaker, one meter in front of subject’s head, was chosen. The noise level was fixed at 65 dB Sound Pressure Level. The speech level was adaptively adjusted according to the number of words identified correctly, starting at 0 dB SNR. The target was approximately the signal-to-noise ratio equivalent to 50% intelligibility. The sentence 2 through 5 were adapted between − 3 dB (‘5 words correct’) and + 3 dB (‘no words correct’), while the sentence 5 through 31 were adapted between − 2 dB and + 2 dB [6]. The SRT was determined by counting the number of words correctly perceived per sentence as a function of the SNR. To get used to the test procedure and to reduce learning effects, the first 20-sentence list was used for training, while second list retained the actual test.

### HINT

#### Recording of sentences

The speech material of the HINT with a female speaker is comparable to that of the HINT with a male speaker. Joiko et al. formed at first 700 sentences of 6–7 syllables in length [17]. Following ICRA recommendations, the speaker for the recordings was a 58-year-old native, non-formally trained German-speaking female [2]. She was instructed to use a clear voice, normal vocal effort, conversational speaking rate and natural articulating for the recording. Her speech was subjectively analyzed by speech therapist’s recommendation and considered appropriate for the recordings. Misspoken sentence or sentences with disturbing noises were repeated twice. The best recordings of the sentences were selected, edited into individual waveform files and rescaled to 16-bit, 24 kHz equal waveform levels using Wavelab Pro 9 digital audio software. The most natural 334 sentences after evaluation by five German native speakers. The average long-term spectrum of the sentences was computed and the masking noise within the same spectrum was synthesized as recommended by Akeroyd et al.

#### Determination of PI function and equalization of sentence difficulty

The scaling and adjusting of sentences RMS levels was performed using the same procedure as recommended by ICRA [2]. The word intelligibility of three 50-sentence-lists at fixed SNRs of − 7 dB, − 4 dB and − 2 dB was used to determine the slope in the area around 75% speech intelligibility. Compared to other languages (Soli and Wong 2008) rather 75% than 70% intelligibility was used for the German HINT, because the PI function of German language was shifted to lower SNR results. 75% are rather close to the recommended reach threshold of 80% word recognition threshold for optimization by ICRA. The test was done in sound field conditions (0° azimuth), with fixed noise level at 65 dB (recommended by ICRA) and using the Speech Utility Program. A Graeco-Latin-Square-Design was used to ensure that each of the three lists was used with each of the three SNR values and in every possible order. The mean percent intelligibility score of the tested SNRs was used to generate the PI function. A change on speech intelligibility of 10% per dB was seen in comparative language by Soli und Wong (2008) and was expected for this study [30]. The slope of the function by the female speaker is comparable to the slope of the male speaker (see below). Contrary to the recommendations by ICRA, the PI function of this study was only used to evaluate the relationship between the percent word intelligibility and change in SNR and to adjust the RMS level of the sentence to achieve approximately the same word intelligibility scores at fixed masking noise levels [2]. Sentences with intelligibility scores other than 70% were adjusted. If the score was higher than 70%, the RMS levels went down, if the score was lower, the level went up. The 240 male HINT sentences with the smallest RMS level adjustments were arranged into 24 ten-sentence lists. The formation of the twelve-20-sentence lists was done by pairing the highest and lowest mean SRTs of the ten-sentence-list measured adaptively by six subjects under in noise and sound field conditions. Joiko et al. shown the non-significant difference in the difficulty of the 20-sentence lists when evaluated by ANOVA [17]. A female speaker’s twelve-20-sentence-lists belong to the same matter as the male HINT’s sentence and lists.

#### Determination of norms

The measurements to determine the norms were conducted binaural and with headphones to exclude room acoustics effects by sound fields. The sound location of the speech signal was constantly from the front (0° azimuth), while fixed noise level was located from the front (0° azimuth; NF), the right (90 azimuth, NR), the left (270 azimuth, NL) or no noise (quiet). The process of generating source located speech and noise signals for headphone presentation was done by processing and stimulating both signals through digital filters using head-related transfer functions (HRTF). The HRTF is associated with different source locations to produce appropriate interaural time and level differences (ITDs and ILDs) for bilaterally equal presentation level. The HRTF is equal for all languages. More details are mentioned in prior publications [8, 17, 30]. The test in sound field without noise was done for calibration procedure. The noise level was kept constant at 65 Db, while the speech level was adaptively varied. The speech level of the first 4 sentences varied in 4 dB steps of the remaining 16 sentences in 2 dB steps. If the entire sentence was repeated correctly, the SNR level of the next sentence was decreased, if the sentence was repeated incorrectly, the level was increased. The starting speech level in Quiet was about 20 dB, the SNR of the NF condition was about 0 dB and of the NR and NL condition about − 15 dB. The SRT was determined by average SNR level of the sentences 5–20 and the presumed SRT of unpresented sentence 21, which was determined from the presentation level to the 20th sentence.

The measurement to determine the norms for the female speaker were part of the comparative measurement with the male speakers HINT and OLSA. Half of the subjects started with the HINT (from that, half with the female and half with the male speaker), while the other half started with the OLSA. The first measuring of the condition Noise Front (NF) was done for practice and to get familiar with the test procedure. The conditions NF (practice), NF (test), NR, NL and Quiet (all measurements using headphones) and Quiet (in sound field) were completed once for each speaker. The resulting test protocol includes available 12 lists. Six of them were part of the measurements to evaluate the female HINT, the other six lists were part of the comparison measurements with the male HINT. Each sentence and list was used once per subject. A balanced Graeco-Latin-Square-Design for the assignment of test condition and list numbers were developed for 12 subjects. Each protocol was run twice, once with female and once with male subject. The order of the sentence was randomized by the test software. The subjects’ individual SRTs were calculated. The means and standard deviations for the 24 subjects were calculated to define the norms. The Noise Composite Score (NCS) (2*NF + NR + NL /4) was used to provide a single overall measure of speech intelligibility in noise [30]. The international comparison of the German HINT was based on the H-Score and the percentiles determined from the mean and standard deviation of the SRT [30].

## Results

### Subjects

Thirty-four subjects participated in the study. Ten of them (6 males; 4 females, mean age 26.2 ± 1.83 years) were participants in the measurement to determine the PI function. Twenty-four normal hearing subjects (12 males, 12 females, mean age 26.8 ± 3.4 years; pure-tone hearing threshold levels ≤ 20 dB hearing level for frequencies 125–8000 Hz were participants in the task to determine the norms.

### Acoustic–phonetic analysis of female and male speaker of HINT

The acoustic software Praat [3] and some phonetic scripts [24] were used for the acoustic–phonetic analysis of both HINT speakers voices. The mean fundamental frequency F0, the minimal and maximal F0, the mean F0 range and the speaking rate were calculated for each speaker by *n* = 36 randomly selected sentences (3 sentence each list). In addition, the average formant values F1 and F2 for *n* = 12 randomly selected vowels /a:/, /u:/ and /i:/, the F1 and F2 range of the mentioned vowels and the vowel triangle A(_vocal diagram_) were determined. Table 1 shows the F0 values [Hz] for the female and male speaker.

**Table 1:** Fundamental frequency for the female and male speaker of the German HINT

Frequency [Hz]	Female	Male
Mean ± SD	179 ± 11	105 ± 4
Min	125	80
Max	245	143
Range	119	63

A significant difference (t test for independent samples/Mann–Whitney test *p* < 0.001) within a large effect size between the mean F0 (*r* = 0.98) and the F0 range (*r* = 0.86) was calculated. The mean F0 and the F0 range for the female speaker’s voice are significant higher and wider compared to the male speakers’ voice.

The mean speaking rate of the female speaker is about 287 syllables per minute and of the male speaker about 251 syllables per minute (*n* = 36). The Mann–Whitney test shows a significant difference in the speaking rate of both speakers *p* < 0.001 (mean effect sizes, *r* = 0.39). The average formant values F1 and F2 of the vowels /a:/, /u:/ and /i:/, the size of the vowel triangle and the range of the formants F1 and F2 of the mentioned vowels of the female and male speakers’ voice are shown in Table 2.

**Table 2:** Means of the formant frequency values F1 and F2 of the vowels /a:/, /u:/ and /i:/, the size of the vowel triangle and the F1 and F2 range for the female and male HINT speakers voice including *p* values

Frequency [Hz]	/a:/ (*n* = 12)		/u:/ (*n* = 12)		/i:/ (*n* = 12)		A_(vocal triangle)_ [Hz^2^]	F1 range	F2 range
	F1	F2	F1	F2	F1	F2			
Female	942	1455	331	797	334	2577	542.803	748	2206
Male	694	1240	362	1217	276	2062	141.259	540	1731
*p* values	< 0.001***	< 0.001***	0.319	0.039*	< 0.001***	< 0.001***			

*p < 0.05; **p < 0.01; ***p < 0.001

**Fig. 1:**
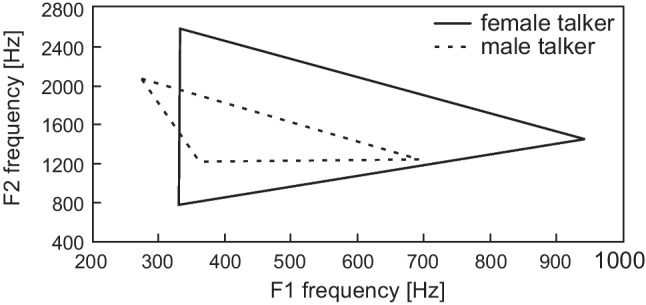
Vowel triangle with corresponding F1 and F2 formant frequency of the female and male HINT speakers voices

A significant (*p* values < 0.05) and large effect between both voices was calculated for the formant values F1 and F2 of the vowels /a:/ and /i:/ (*r* = 0.90 (F1) and *r* = 0.84 (F2), for /a:/and *r* = 0.75 (F1) and *r* = 0.88 (F2), for /i:/). The formants F1 and F2 of both vowels of female HINT speakers’ voice are placed at significant higher frequencies. The frequency of the formant F2 of the vowel /u:/ of the male speaker’s voice is shifted to significant higher levels (mean effect sizes, *r* = 0.42). The F1 and F2 range of the mentioned vowels and the size of the vowel triangle are shown in Table 2 and Fig. 1. They are larger by female speaker’s voice than male speakers’ voice (absolute data difference).

### HINT with female speaker

#### PI function

**Fig. 2:**
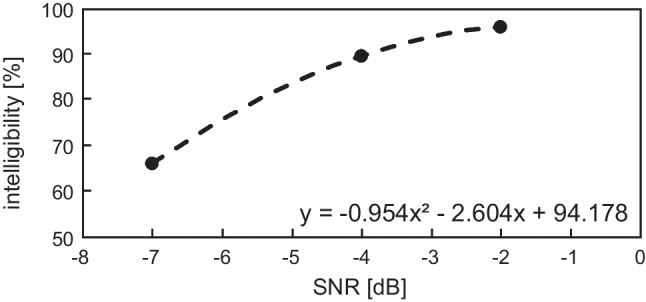
PI function with the average intelligibility scores of tested SNRs (spots) and the function equation using second-order polynomial trendline (dashed trace) is displayed

Figure 2 shows the PI function of the average intelligibility scores at the mentioned fixed SNRs (− 7 dB, − 4 dB and − 2 dB). The PI slope of the second-order polynomial trendline is about 8.84% per dB in the area around 75 intelligibility (Fig. 2).

#### Norms

The normative values (mean and standard deviation of the SRT) for the HINT with a female speaker of the conditions NF, NR, NL, NCS and Quiet under headphones are shown in Table 3.

**Table 3:** Mean and SD of the speech reception thresholds (SRT) of the tested conditions for female and male HINT speakers and the norms from Joiko et al. 2020 for HINT with a male speaker

	Noise front		Noise right	Noise left	NCS	Quiet
	Training mean ± SD [dB SNR]	Test mean ± SD [dB SNR]	Mean ± SD [dB SNR]	Mean ± SD [dB SNR]	Mean ± SD [dB SNR]	Mean ± SD [dB]
Female talker	− 5.46 ± 0.71	**6.07 ± 1.02**	− 14.19 ± 1.15**	− **14.20 ± 1.55**	− **10.12 ± 0.54**	**19.09 ± 2.40**
Male talker	− 5.60 ± 1.04	− 5.81 ± 0.75	− 14.13 ± 0.80	− 14.73 ± 1.01***	− 10.13 ± 0.68*	18.28 ± 2.44*
Norms from Joiko et al. (2020)		− 6.00 ± 0.80	− 13.60 ± 0.90	− 13.70 ± 0.70	− 9.88 ± 0.50	19.20 ± 2.60

Bold values indicate SRT values to define the norms for HINT with a female speaker. Significant results comparing the mean SRT of female and male HINT with the norms from Joiko et al. 2020 [17] are marked with *p < 0.05; **p < 0.01; ***p < 0.001

The results of the conditions training and test, NF of both speakers were compared with paired *t* test. A significant difference was observed for the female speaker (*p* = 0.022, unilateral) but not for the male speaker (*p* = 0.18, unilateral). Completing one 20-sentence list seems to be sufficient for normal hearing subject to become familiar with the test procedure of the HINT with a female speaker (small effect size, *d* = 0.43).

Table 4 shows the headphone norms and the intelligibility change using the calculated H-Scores and the percentiles. Scoring results below the mean score (50th percentile) are represent within lower individuals’ intelligibility, results above within higher intelligibility. The rows threshold and intelligibility change defining the expected difference (in dB or dB SNR and percent) between the mean score and the percentile (column) for the conditions.

**Table 4:** Norms for the German HINT with a female speaker measured via headphones with corresponding H-Score and percentiles [*n* = 24]

Percentile	2.5	5	10	20	25	30	40	50	60	70	75	80	90	95	97.5
H-Score	51	59	68	79	83	87	94	100	106	113	117	121	132	141	149
Quiet (Mean = 19.1 SD = 2.4)															
SRT [dB]	23.8	**23.0**	22.2	21.1	**20.7**	20.3	19.7	**19.1**	18.5	17.8	**17.5**	17.1	16.0	**15.1**	14.4
Intelligibility change (%)	− 47%	− **39%**	− 31%	− 20%	− **16%**	− 13%	− 6%	**0%**	6%	13%	**16%**	20%	31%	**39%**	47%
Noise Front (Mean = 6.1 SD = 1.0)															
SRT [dB SNR]	− 4.1	− **4.4**	− 4.8	− 5.2	− **5.4**	− 5.5	− 5.8	− **6.1**	− 6.3	− 6.6	− **6.8**	− 6.9	− 7.4	− **7.7**	− 8.1
Intelligibility change (%)	− 20%	− **17%**	− 13%	− 9%	− **7%**	− 5%	− 3%	**0%**	3%	5%	**7%**	9%	13%	**17%**	20%
Noise Right (Mean = 14.2 SD = 1.2)															
SRT [dB SNR]	− 11.9	− **12.3**	− 12.7	− 13.2	− **13.4**	− 13.6	− 13.9	− **14.2**	− 14.5	− 14.8	− **15.0**	− 15.2	− 15.7	− **16.1**	− 16.4
Intelligibility change (%)	− 23%	− **19%**	− 15%	− 10%	− **8%**	− 6%	− 3%	**0%**	3%	6%	**8%**	10%	15%	**19%**	23%
Noise Left (Mean = − 14.2 SD = 1.6)															
SRT [dB SNR]	− 11.2	− **11.7**	− 12.2	− 12.9	− **13.2**	− 13.4	− 13.8	− **14.2**	− 14.6	− 15.0	− **15.2**	− 15.5	− 16.2	− **16.7**	− 17.2
Intelligibility change (%)	− 30%	− **25%**	− 20%	− 13%	− **10%**	− 8%	− 4%	**0%**	4%	8%	**10%**	13%	20%	**25%**	30%
Noise Composite Score (Mean = − 10.1 SD = 0.5)															
SRT [dB SNR]	− 9.1	− **9.2**	− 9.4	− 9.7	− **9.8**	− 9.8	− 10.0	− **10.1**	− 10.3	− 10.4	**− 10.5**	− 10.6	− 10.8	− **11.0**	− 11.2
Intelligibility change (%)	− 11%	− **9%**	− 7%	− 5%	− **4%**	− 3%	− 1%	**0%**	1%	3%	**4%**	5%	7%	**9%**	11%

### Effect of the speaker’s gender on the first HINT measuring

The comparison of the SRT of the first HINT measuring depending on the gender of the speaker was done by the *t* test for independent samples. The *p* values for all conditions were not significant (*p* ≥ 0.05). The non-significance result of the speaker’s gender on speech intelligibility of the first HINT measurement was supported by small absolute differences: the mean SRTs (in SNR) for NF training were about − 5.51 dB (female speaker) and − 5.33 dB (male speaker), for NF test about − 6.29 dB (female speaker) and − 5.84 dB (male speaker) and for NCS about − 10.35 dB (female speaker) and − 10.13 dB (male speaker). In addition, a multi-factorial ANOVA of the threshold results was conducted as between speakers’ and subjects’ gender. Again, *p* values were not significant (*p* ≥ 0.05). There are no significant effects of speakers’ and subjects’ gender on the speech intelligibility scores of the first HINT measurement between the female and male HINT speakers.

### Comparison with the norms of German HINT with a male speaker

The norms of the HINT with the female and the male speaker are represented in Table 3 [17]. The norms for the HINT with the female speakers are generally at lower SRTs values than the norms for the HINT with male speaker but without any statistically significance (*p* values > 0.05) except for the condition noise right (*p* = 0.02). The comparison of the resulting SRT of both measurements with the male speaker shows comparable results. The intelligibility scores of the conditions NR (*p* = 0.003), NL (*p* < 0.001) and the NCS (*p* = 0.04) within this study by the male speaker were significant lower than the norms my Joiko et al. [17]. The standard deviations for the norms of both speakers are generally in the same absolute range (shown in Table 3).

### Comparison of the speech intelligibility of HINT and OLSA

**Fig. 3:**
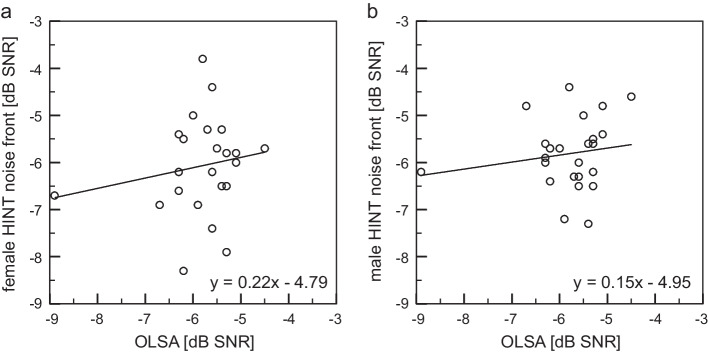
Scatter plot and correlation equation between OLSA test and HINT noise front test for **a** female HINT and **b** male HINT speaker

The mean threshold of OLSA test condition was − 5.8 dB SNR and, therefore, 1.3 dB higher compared to the norms by Wagener et al. about − 7.1 dB SNR [33]. The difference is statistically high significant (one sample Wilcoxon Test, *p* < 0.001). Figure 3 shows the non-significant correlation (including the correlation coefficient *r* and the *p* values > 0.05) between the resulting SRT values of the condition OLSA test and the conditions NF with the female and male speaker.

**Fig. 4:**
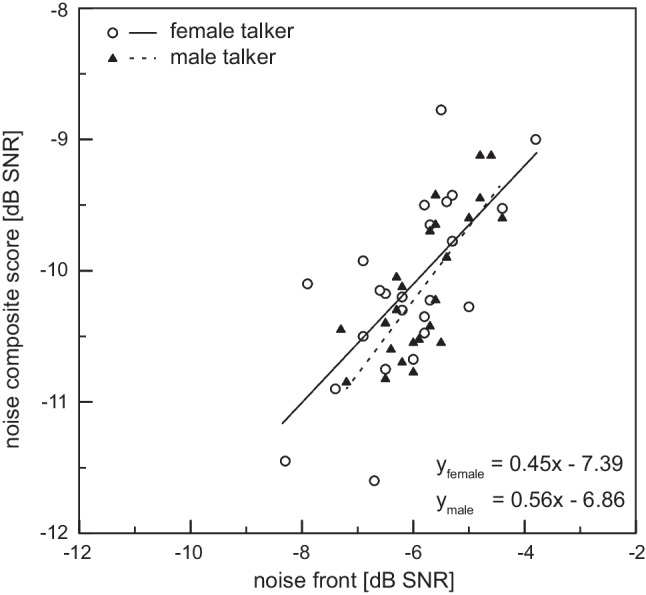
Scatter plot and correlation equation between the test condition noise front and the calculated noise composite score for female and male HINT speaker

The Spearman correlation coefficient was used to investigate the influence of speakers’ gender on the thresholds of the HINT and OLSA. Figure 4 shows the strong, concordant and significant correlation between the condition NF (Test) and the calculated NCS for both speakers (*p* = 0.001 for female and *p* < 0.001 for male speakers’ HINT). The Spearman correlation coefficient was *r*_s_ = 0.63 for the female speaker and *r*_s_ = 0.74 for the male speaker.

A significant correlation *p* = 0.024 and *r*_s_ = 0.46 (mean effect size) was calculated for the SRT of the condition NF between the female and male speaker. Lower SRT values by female speakers HINT are accompanied by lower SRT values by male speakers HINT and vice versa. No significant correlation was calculated between the SRT of the HINT and the OLSA (*p* values higher than 0.05). Finally, the Mann–Whitney Test was conducted to evaluate the effect of subjects’ gender on the SRT of HINT and OLSA. *p* values were not significant (*p* ≥ 0.05). There are no significant effects of a subject’s gender on the speech intelligibility by HINT and OLSA.

## Discussion

The development of German HINT with a female speaker realizes the most important recommendation of the ICRA for the development of multilingual speech tests using the female speaker [2]. The use of the female voice overcomes the only limitation in the conceptual model of the HINT in other languages using male voices [29]. Furthermore, female HINT is suitable for children from the age of 6 years due to its simple sentences and vocabulary and the advantage of female voices for children’s speech test [28].

The German HINT with a female speaker was developed in the same manner as the German HINT with a male speaker [17]. The recorded speech material and the assignment of the sentence to the list of male and female HINT are equal and comparable. In contrast to the ICRA, the female HINT used fixed points of the PI function to calculate the relationship between the SNR change and the percent intelligibility word scores and to adjust the sentence RMS levels. The ICRA recommended to use the entire PI function to equalize the sentences and to form homogeneous test lists. The equalization of the sentence of the HINT is done using a single point on the discrimination function. Furthermore, while ICRA recommended adaptively 50%- or 80%-word scoring methods, the SRTs by HINT are measured using adaptively 50% correct sentence scoring [2, 17]. The focus of this study was on the influence of Speakers sex on speech intelligibility. No further measurements were done by female HINT about the variability across the sentence-to-list-matching taken over by male HINT.

### Interpretation of the norms of HINT with a female speaker and comparison with the data by HINT with a male speaker and OLSA

According to Brand and Kollmeier, standard deviation values of 1 dB SNR are important to achieve high validity of Speech-in-Noise-Tests [6]. The standard deviations of the norms of the German HINT with a female speaker are in the mentioned range about 0.54 dB (NCS), 1.02 dB (NF), 1.15 dB (NR) and 1.55 dB SNR (NL) and comparable with the SD for the German HINT with a male speaker (NF 0.8 dB, NR 0.9 dB, NL 0.7 dB and NCS 0.5 dB SNR). The average standard deviation in Quiet are lower but still comparable between both speakers (female 2.4 dB, male 2.6 dB). Expect the condition noise right, there was no significant difference between the norms by the female speaker and the norms by Joiko et al. for the male speaker. The speech material including sentence-list-classification of both tests and the studies included subjects are nearly equal. The subjects were young (mean age 26.8 years, Joiko et al. 26.7 years), mostly students and normal hearing listeners (pure-tone hearing threshold levels around 6 dB) [17]. Similar to the results found by Yoho et al. the homogeneity of the subjects seems to be responsible for the mentioned non-significant effects between the female and male HINT and, furthermore, for the non-significant effect of the subjects’ gender on the speech intelligibility score [37]. Unnoticed interindividual subject difference could be responsible for the significant difference between the SRT results of the conditions NR by the female speaker and NR, NL and the NCS by the male speaker of this study compared to the norms from Joiko et al. (NR: − 14.19 (female norms) and − 14.13 (male speakers results) versus − 13.60 dB SNR (male norms), NL: − 14.73 versus − 13.70 dB SNR and NCS: − 10.13 versus − 9.88 dB SNR) [17]. Furthermore, Joiko et al. calculated the male HINT norms by 40–91 SRT results contrary to the 24 SRT data for determination of the norms of the female HINT [17].

Nevertheless, the threshold norms for the female speaker are generally comparable to the norms for the male speaker. Using a second order polynominal trendline, the slope of the Performance intensity function by female HINT is about 8.84% per dB and by male HINT about 9.8% per dB. The reduced slope for the female speaker could be the consequence of the ‘fixed final HINT sentence from selected 334 natural sentences. Using the same 240 sentences for female HINT was more important than eliminating the ‘poorest’ and ‘best’ intelligible sentence resulting by the slope of the PI function. No adjustments on the selected sentences by female sentences material could be made. The following adjustment of the RMS level of the final female HINT sentences was done based on the results for the female PI measurements.

Both PI slopes are lower than the average slope of the thirteen other languages by Soli and Wong about 10.3% per dB but within the range of the first SD around the mean [30]. The German PI slopes were calculated as a part of the measurements to evaluate the difficulty of the sentences. Furthermore, the German slopes were calculated based on word scoring, whereas the slopes of the other languages were calculated based on sentence scoring protocols. Nevertheless, the PI function German female and male HINT are comparable to the slope of the mentioned thirteen other languages. The slopes are still lower than OLSA’s introductory slope of about 17.1% per dB. Larger changes in speech sound pressure or SNR are required by HINT compared to OLSA to achieve differences in speech intelligibility [33].

Harianawala et al. compared the slope of the PI function of the American matrix test and the American HINT determined by fixed SNRs including ten subjects with hearing aids. Contrary to the slopes by normal hearing subjects, the slope of the HINT was steeper (∼ 14% per dB) than the slope of the matrix test (11% per dB). The HINT seems to have small advantages by detecting differences in speech intelligibility resulting from variations in hearing aid processing [14]. Further studies for detailed results of the PI slope for the German HINT including normal hearing subjects as well as subjects with hearing aids are recommended.

In their study, Soli and Wong compared the SRT means and standard deviations of thirteen languages [30]. The average SRT for other languages were − 3.9 dB SNR (NF), − 11.2 dB SNR (NR), − 11.3 dB SNR (NL) and − 7.6 dB SNR (NCS) (refer to Table 1, Soli and Wong 2008) [30]. The noise condition norms of the German HINT with a female speaker (shown in Table 3) are significantly lower (better) than the average thresholds for other languages [17, 30]. Hochmuth et al. investigated the speech recognition of matrix sentences spoken by German/Russian and German/Spanish bilingual speakers [16]. The authors found both language and speaker specific effects for the German/Spanish bilingual speakers. German SRTs were generally 2–4 dB lower than Spanish SRTs. Specific articulation and grammatical features and the structure of the Spanish language could responsible for these effects [16]. Additional studies using bilingual HINT speakers are useful to evaluate the SRT difference made between German and other languages and to examine possible specific features and characteristics of the German language. Nevertheless, speaker-specific characteristics affected speech intelligibility in noise more than language-specific characteristics [16, 30]. Especially conditions in Noise seems to be relevant. The mean values of the conditions in Quiet of German female and male HINT are in the range of the mean SRTs between 15.3 dB and 25.9 dB (mean 18.4 ± 3.5 dB of the other languages) across other languages [30].

A significant interaction between the SRT values of the conditions NF and NCS were expected, because the score is calculated by threshold results of the investigated noise conditions. The significant correlation between the threshold values of the conditions NF by the female and male speaker supports the observation about the non-significant influence of speaker’s gender on speech intelligibility. Instead, the speech material seems to have a relevant impact on speech intelligibility scores showing non-significance correlation between the SRT results of HINT and OLSA. The results by OLSA must be evaluated talking into account of the test method and setting used. The results of the OLSA (mean − 5.80 dB SNR) differ significantly from the norms by Wagener et al. about − 7.1 dB SNR. The results of the present study were evaluated under headphone conditions and after a training session of 30 sentences compared to the data by Wagener et al. who recommended and used 6 list per 20 sentences for training and sound field conditions [33]. Results in sound field conditions are influenced by the individual acoustics effects of the audiology test environment [30]. The aim of the study was to develop norms for the German HINT with female speakers and to compare these norms with the German HINT with male speakers under headphone presentation. To be comparable and to eliminate variable effects of room acoustics, a headphone setting was also used by OLSA. Nevertheless, the completion of the training procedure of the OLSA by one list compared to the mentioned six lists by Wagener could be responsible for the significant difference between the resulting OLSA mean values compared to the norms by Wagener et al.. The HINT measurements were performed by completing the training session using one 20-sentence-list. Similar to the results by the Swedish HINT by Hällgren et al., a short training session seems to be sufficient for normal hearing subjects to get familiar with the test procedure [13]. The measurements of each subject included in total 12 lists per 20 sentences. A high level of concentration was required of the subjects during the study which lasted about 2 h. As a consequence, shorter training sessions were used. Nevertheless, the comparability of the resulting SRT values of OLSA and HINT must be considered in the context of their individual test environment, the difference within the test procedure and the scope of training.

The most important variable of speech-in-noise tests concerns the speech material (e.g., numbers, words, rhyme and sentence) and in the present study focuses specifically on sentence structure and vocabulary. The limited vocabulary (50 words) of the OLSA being referred to as a ‘semi-open’ test. Repeated words could be recognized and are easier to guess. Mentioned influence could distort the results like moving the SRT to lower (better) SRT values. The phoneme distribution of the OLSA base list represents the phoneme distribution of the German language. The OLSA Test was developed in 1999 [33–35]. It’s reference data for the phoneme distribution of the utilized words and names are from the 1970s are outdated [34]. Especially some used names are outdated and, therefore, easy to remember for younger subjects. The phoneme distributions of 2019 generated HINT sentence within each of the twelve 20-sentence-lists did not differ significantly [17]. Further studies are planned to investigate the ICRA recommendations about language and contextual skills and adult specific phoneme distribution of the German language [2].

The vocabulary size of the HINT words is more extensive compared to the OLSA. Nevertheless, matching with childlex database limits the ability of speech materials using vocabulary of 6–8-year-old children [17]. Further studies are needed to verify the German HINT with a female speaker within younger subjects as well as normal hearing adults to evaluate a test–retest reliability measurement.

In addition, cognitive effects and the working memory capacity influences speech intelligibility. Rudner et al. have shown that these effects are larger by Matrixtest than everyday sentences test like the HINT [27]. Matrixtest are semantically unpredictable and less redundant. Constrained structures and the content of Matrixtests make guessing more difficult compared to meaningful and simple HINT sentences. With age, the linguistic and context-related skills increase, while working memory capacity decreases [27]. In clinical routine, speech-in-noise-tests are used to evaluate changes in individual speech recognition that may lead to an indication for hearing aids. Mean age of clinical tested subjects is, therefore, mostly older than 26.8 years. Uslar et al. evaluate within their study the influence of three different types of linguistic complex sentences on speech reception in noise for younger and older subjects. Even if their study shows less relevance of linguistic complexity on speech reception in noise, the authors are sure about the influence of linguistic complexity as well as the age and the hearing conditions of the subjects. Further comparative HINT and OLSA studies should include different groups of subjects (e.g., younger and older subjects) to assess the influence of subject age and the linguistic complexity across the used sentences on speech recognition [32]. The comparison of the results of the first and the second measurements of OLSA and HINT showed larger training effects for the OLSA (absolute difference between the mean SRT of the first and second measurement: OLSA: 0.72 dB SNR, female HINT: 0.61 dB SNR, male HINT: 0.21 dB SNR). The HINT seems advantageous to assess speech recognition more quickly and without extensive training sessions, but in context of its limited number of lists and possible learning procedures with repeated use.

Following HINT studies should be done to investigate the effect of repeated lists across test sessions on the speech intelligibility score. After completion of the training procedure, the OLSA seems suitable for frequent measurements in the hearing aid fitting process because of its random repeatability. Supporting the first notices by Hällgren et al., the test environment, the test methods including speech material and training effects and the included subjects’ have an influence on the speech recognition in noise [13].

### Effect of speakers’ gender on speech intelligibility score

Further aim of the present study was to investigate the effect of female and male speakers voice on speech recognition threshold of normal hearing subjects. A lot of studies investigated the influence of speakers’ gender and its acoustic phonetic characteristics on speech intelligibility. Hazan and Markham (2004) [15] and Bond and Moore (1994) [4] showed that longer word duration and slower speaking rates are correlating with better speech intelligibility scores. There was no correlation with the mean F0 range in the study by Hazan and Markham [15]. The mean speaking rate of both male speakers in the present study is significantly lower (233 syllable/ min, OLSA and 251 syllable/ min, male HINT) compared to female speakers who speak about 287 syllable/min [34]. Nevertheless, the reached SRT values did not differ between the female and male HINT speakers. Similar results were found by Bradlow et al. (1996) by investigating sentence intelligibility in quiet. A slower speaking rate and the mean F0 did not correlate with better speech intelligibility scores [5]. Hazan and Braida showed that speakers’ timing, the precision of articulation and the long term spectra in the 1–3 kHz region correlate significantly with better intelligibility of female speakers [15]. Krause and Braida emphasize the importance of a clear voice compared to a slower speaking rate. By subjective judgment, the female HINT voice seems to be more clear and phonetical accentuated than the male voice. Nevertheless, the SRT norms do not differ. The influence of the “intrinsically clear” speaking style on speech recognition scores seems to be less important in the present study compared to the data by Krause and Braida [22]. Further studies should investigate the mentioned subjective findings about HINT speakers speaking style.

Bradlow et al. (1996) found that female speakers were significantly more intelligible than male speakers using sentences in quiet conditions. The authors explained their data by higher F0 range and wider dispersion of the phonetic vowel space of female voices compared to male voices [5]. The results by Bond and Moore and Hazan and Markham supports the relevance of a wider vowel space for better speech intelligible scores [4, 15]. The mean F0 range and the dispersion of vowel space (F1 and F2 values of the vowels /a:/ and /i:/, F2 value of /u:/ and the difference between F2 and F1of /i:/) differ significantly between female and male voices by wider range of female speaker. The vowel size as well as the F1 and F2 range are larger at female speakers’ voices compared to male speaker’s voices (refer to Table 2). Nevertheless, both speakers’ intelligibility scores did not differ significantly. The mentioned acoustic parameters seem to have no relevant influence on the SRT results in the present study. Still, the statement must be interpreted in the background about the limited numbers of analyzed vowels (*n* = 3) and sentence (*n* = 36) and the linguistic complexity (refer to Uslar et al.) based on phoneme.

In 2013 Ahrlich developed the German female Matrix test. The mean SRT is about – 9.3 dB SNR compared to the normative SRT by Wagener et al. which is about – 7.1 dB SNR. Despite an extensive analysis of the speech-acoustic parameters by six different speakers (female OLSA; male OLSA and four bilingual speakers—two female, two male whose speech material and SRT values originate from Hochmuth et al. 2013), the authors could not determine a clear connection between the resulting mean SRT and discussed acoustic parameters (mean F0, mean F0 range, vocal range by /a:/, /i:/ and /u:/, vowel triangle, F1-/ F2-range, speaking rate). Similar to this study, the authors could not present any correlation between the speakers’ gender and the resulting SRT values [1].

In conclusion, the present study supports the illustrated inconsistency of the mentioned studies. There are no specific acoustic–phonetic characteristics that force higher intelligibility scores. Furthermore, especially the presentation modus—namely, within and without noise, has an important influence on speech intelligibly score and speakers intelligibility as well. Different speakers use different combinations and strategies of the mentioned acoustic–phonetic parameters to achieve high intelligibility scores. High interindividual variabilities in the speaker’s speaking style obtain the difficulty that a single speaker may never be fully representative of his or her current gender as a whole The female voice of the German HINT had a rather lower mean F0 about 179 Hz compared to literatures mean F0 about 200 till 220 Hz [31]. Despite a large difference between the mean F0 of the female and male HINT speakers’ voice (105 Hz, male and 179 Hz female voice) the mentioned lower mean F0 of the female voice offers a possible explanation for the equality and non-significance difference between both HINT norms of three of the four tested conditions (NF, NL and Quiet) and the calculated NCS.

Calculating speech intelligibility scores by a single speaker while knowing about the diversity of voices and their differences in acoustic phonetic characteristics in the everyday acoustic environment seems to be difficult. Instead, speech intelligibility should be reviewed by comparing results of different speakers of different genders. Kelly et al. developed in 2017 a mixed gender, multi-speaker matrix sentence test in Australian English. Ten speakers, five women and five men, some of them professional actors and some amateur actors, were part of the recording process. The norms and slope of the multi-speaker matrix sentence test were comparable to reference values obtained from other matrix test involving single speakers (e.g., German and Swedish matrix test [18]). Speech recognition scores investigated by speech-in-noise-tests within both speakers are more realistic presentations of the everyday acoustic environment, although there was no significant reference of the HINT talker gender for the norms, except of one condition.

Finally, it is recommended and necessary to integrate further speech-in-noise-tests, especially tests with female speakers in clinical audiological assessment. Referring to Harianawala et al. (2019), the clinical decision for the use of one of the multitudes of mentioned speech-in-noise-tests will depend on different variables and factors. Test-related factors are about the indication and aim of the test, about the including subjects and their linguistic and cognitive skills, the availability of the speech material and the test time, about the experience within the recommended test and the expected or known power of speech intelligibilities disorder [14].

## Conclusion

The purpose of this study was to develop the German HINT with a female speaker and to compare the speech recognition score obtained with the German HINT with a male speaker and German Matrix sentence-test (OLSA). The German HINT with a female speaker is suitable addition to the known Speech-in-noise-tests with a male speaker. The evaluated norms by HINT with a female speaker are comparable to the norms by HINT with a male speaker. Despite high interindividual variability of acoustic–phonetic characteristics between the female and male speaker, speech intelligibility scores must rely independently of speaker’s gender but at the same time dependent on the speech material used in the test.

## Abbreviations

F0: Fundamental frequency
F1, F2: Formant frequencies
HINT: Hearing in noise test
ICRA: International Collegium of Rehabilitative Audiology
NF: Noise front
NL: Noise left
NR: Noise right
NCS: Noise composite score
OLSA: Oldenburg sentence test
PI function: Performance intensity function
SD: Standard deviation
SNR: Signal-to-noise-ratio
SRT: Speech reception threshold
